# Serum alpha-mannosidase as an additional barrier to eliciting oligomannose-specific HIV-1-neutralizing antibodies

**DOI:** 10.1038/s41598-020-64500-8

**Published:** 2020-05-05

**Authors:** Jean-François Bruxelle, Tess Kirilenko, Quratulain Qureshi, Naiomi Lu, Nino Trattnig, Paul Kosma, Ralph Pantophlet

**Affiliations:** 1 Faculty of Health Sciences, Simon Fraser University, Burnaby, British Columbia V5A1S6 Canada; 2 Department of Molecular Biology & Biochemistry, Simon Fraser University, Burnaby, British Columbia V5A1S6 Canada; 3 Department of Chemistry, University of Natural Resources and Life Sciences, Vienna, A-1190 Austria; 4 AbCellera Biologics Inc., Vancouver, British Columbia Canada; 5 Department of Chemical Biology and Drug Discovery, Utrecht University Utrecht, The Netherlands

**Keywords:** Carbohydrates, Conjugate vaccines, Antibodies, Glycobiology

## Abstract

Oligomannose-type glycans on HIV-1 gp120 form a patch that is targeted by several broadly neutralizing antibodies (bnAbs) and that therefore is of interest to vaccine design. However, attempts to elicit similar oligomannose-specific bnAbs by immunizing with oligomannosidic glycoconjugates have only been modestly successful so far. A common assumption is that eliciting oligomannose-specific bnAbs is hindered by B cell tolerance, resulting from the presented oligomannosides being sensed as self molecules. Here, we present data, along with existing scientific evidence, supporting an additional, or perhaps alternate, explanation: serum mannosidase trimming of the presented oligomannosides *in vivo*. Mannosidase trimming lessens the likelihood of eliciting antibodies with capacity to bind full-sized oligomannose, which typifies the binding mode of existing bnAbs to the oligomannose patch. The rapidity of the observed trimming suggests the need for immunization strategies and/or synthetic glycosides that readily avoid or resist mannosidase trimming upon immunization and can overcome possible tolerance restrictions.

## Introduction

Development of an effective HIV vaccine is still a high priority^1,2^. One of the central challenges remains the creation of a formulation that elicits broadly neutralizing antibodies (bnAbs) to the HIV envelope spike (Env). Six sites on Env are now recognized as vulnerable to bnAbs^3^, thus constituting vaccine targets. One of these sites is a conserved patch of oligomannose-type glycans on gp120. However, attempts to elicit bnAbs to this patch by immunization have not been fruitful.

The prevailing hypothesis is that tolerance mechanisms hinder the elicitation of oligomannose-specific bnAbs. Although the occurrence of oligomannose-type glycans is rare on healthy human tissue and cells^4^, a few human plasma glycoproteins do seem to sparsely express oligomannose-type glycans under normal physiological conditions^4^. The occurrence of these oligomannose-type glycans, even though not abundant, may be sufficient to limit the frequency of naïve B cells with receptors capable of binding oligomannose or render such ‘self-reactive’ B cells anergic^5^. Indeed, tolerance mechanisms are known to limit the repertoire frequency of naïve B cells with capacity to bind host glycan structures^6^. The identification of B cells with autoreactive signatures in HIV-infected individuals^7–9^, including those from whom oligomannose-specific bnAbs have been recovered^9^, has strengthened the notion that eliciting bnAbs, at least those to nominal self-glycans, may require tolerance restrictions to be overcome in some manner.

Most attempts to elicit oligomannose-specific bnAbs have involved the use of glycoconjugates with dense oligomannosyl clusters^10–17^. Dense clusters of oligomannose-type glycans are atypical of mammalian host protein glycosylation and thus were once thought suitable for defeating tolerance^18^. However, antibodies elicited by such approaches have been nearly invariably specific for oligomannoside substructures, rather than full-sized oligomannose as occurs on Env. These outcomes are interpreted commonly as a remaining manifestation of tolerance or conformational variances between natural oligomannose on Env and synthetic oligomannoside clusters^18,19^.

One possible alternate explanation for the prevalence of antibodies that are specific for oligomannose substructures—rather than full-sized oligomannose—could be that the elicited antibodies reflect a heretofore unappreciated occurrence *in vivo*: enzymatic trimming of the administered glycosides. This notion was raised in a recent report^20^ describing an attempt to elicit 2G12-like bnAbs in rabbits using multivalently displayed glycopeptide immunogens. The report showed that the resulting antibodies were largely specific for the proximal glycan core rather than the distally located tips of the target glycans^20^. It was suggested that this outcome might be the result of the activity of a mannosidase in (rabbit) serum that trims full-sized oligomannose on administered glycoproteins^21–23^. Serum mannosidase trimming has been reported to occur fairly rapidly (*t*_½_ ~5–6 h)^21,22^; if so, then that would readily decrease the availability of full-sized oligomannosides for recognition by B cells at priming and subsequent antibody affinity maturation in germinal centers^24^. The significance of such enzymatic trimming is substantial given that bnAbs to the oligomannose patch on HIV gp120 are specific for full-sized oligomannose^9,25–28^ and considering that glycans are a major component of the epitope of many other bnAbs.

Here, we report on our own investigation of serum mannosidase as an additional potential hurdle to eliciting oligomannose-specific bnAbs. First, we show that bnAb PGT128, an example of the newer generation of bnAbs to the oligomannose patch, binds substantially worse to a microtiter plate-bound glycoconjugate that has been incubated *in situ* with mammalian sera, with sera from mice and humans notably causing the greatest reduction. Antibody binding is restored when kifunensine, a highly specific alpha-mannosidase inhibitor^29,30^, or EDTA is added to the serum, demonstrating that the loss of antibody binding is due to Ca^2+^-dependent alpha-1,2-specific mannosidase activity. Secondly, and perhaps more significantly, we show that early antibodies produced in animals immunized with a CRM_197_-conjugated glycomimetic of oligomannose^31^ bind better to serum-treated glycoconjugate (i.e., enzyme-trimmed) than to buffer-treated glycoconjugate, suggesting that glycoside trimming indeed occurs *in vivo*. In addition to having obvious implications for the design of glycoconjugates for eliciting oligomannose-specific bnAbs, we show that our findings are also of relevance to efforts employing recombinant envelope glycoproteins.

## Results

### **Bacterially derived glycomimetic of mammalian oligomannose conjugated to protein carrier CRM**_**197**_**retains favorable antigenicity**

**Figure 1:**
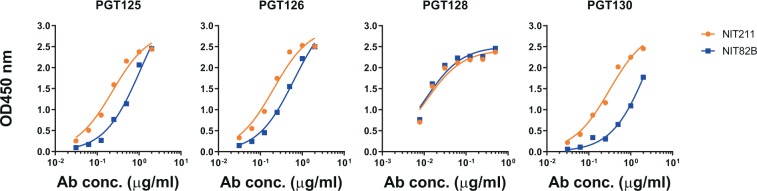
PGT128 and related bnAbs bind the CRM_197_-conjugated mimetic (NIT211) with similar or greater avidity as the BSA-conjugated version (NIT82B). The NIT211 derivative (NIT211_3) used here is loaded at 3.5 glycosides per CRM_197_. NIT82B is loaded at 4.4 glycosides per BSA. The conjugates (63–73 kDa) were coated as solid-phase antigen onto microtiter-plate wells at 5 µg/ml and assayed for recognition by PGT125, 126, 128, and 130. All antibodies were tested as IgGs.

We have reported previously on a synthetic antigenic mimic of mammalian oligomannose^31^, which was designed based on the lipooligosaccharide backbone of an *Rhizobium radiobacter* strain that closely resembles the D1 arm of oligomannose^32,33^ and then synthetically extended to create an analog of the D3 arm of mammalian oligomannose. For practical purposes, the mimetic was conjugated initially to BSA^31^. We subsequently conjugated it to CRM_197_, a more clinically apt carrier protein^34^ that stimulates robust T-follicular helper (Tfh) responses^35–38^. As before^31^, the glycomimetic, equipped at the reducing end with an amine linker, was conjugated to lysine residues present on the protein carrier, via an isothiocyanate intermediate. MALDI-TOF analyses showed that, depending on the batch, 3.5–6.5 glycosides could be conjugated per CRM_197_ molecule (Supplementary Fig. S1). We evaluated the ability of bnAb PGT128 and three related members of the PGT128/130 bnAb family to bind this new CRM_197_ glycoconjugate, which was named NIT211. As shown in Fig. 1, all four bnAbs bind NIT211 at least as good as NIT82B, the initial BSA conjugate^31^.

In summary, our results show that the CRM_197_-conjugate NIT211 reported here presents a reasonable mimic of oligomannose as occurs on HIV Env, as evidenced by reasonably strong binding of bnAb PGT128 and related antibodies.

### **Serum mannosidase trims the CRM**_**197**_**-conjugated glycoside*in vitro***

**Figure 2:**
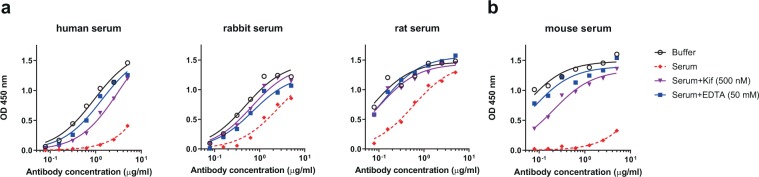
Serum mannosidase trims protein-conjugated oligomannose mimetic *in vitro*. Shown is PGT128 binding to the BSA-conjugated (**a**) or the CRM_197_-conjugated glycoside (**b**) (5 µg/ml) after *in situ* overnight incubation of glycoconjugate-coated ELISA plates with buffer, mammalian serum, serum supplemented with kifunensine (Kif) or serum supplemented with EDTA. All experiments for a given serum were performed on a single assay plate to avoid potential plate-to-plate variability. Shown are representative results from two independent experiments.

In the recent report suggesting that serum mannosidases might be impeding the elicitation of oligomannose-specific bnAbs^20^, the Man_9_-peptide conjugate designed in the study was retrieved for mass spectrometric analysis after incubation in rabbit serum. Results showed that>90% of the Man_9_ was trimmed to Man_6_ within 24 h. In line with the results of that report, we found here using an ELISA format that PGT128 binding to both the BSA-conjugated (NIT82B) and the CRM_197_-conjugate glycoside (NIT211) is substantially diminished after a 24 h-incubation *in situ* with different mammalian sera relative to buffer control (Fig. 2). Notably, the reduction in antibody binding was particularly pronounced following incubation in human and mouse sera compared to rat or rabbit sera. Adding EDTA or kifunensine to these sera restored PGT128 binding (Fig. 2), consistent with the enzymatic activity of a Ca^2+^-dependent alpha-1,2-specific mannosidase^21^.

We performed assays to ascertain that reduced PGT128 binding was due to enzymatic activity in serum and not an artifact of our set up. First, we confirmed that the restoration of antibody binding with both kifunensine and EDTA was titratable (Supplementary Fig. S2); PGT128 binding increased with increasing inhibitor concentration (i.e., increased inhibition of mannosidase directly (kifunensine) or sequestration of the ionic cofactor Ca^2+^ (EDTA)). Second, the reduction in PGT128 binding following incubation of the glycoconjugate with serum could also be lessened in the presence of deoxymannojirimycin (DMJ) (Supplementary Fig. S2), another alpha-mannosidase inhibitor. These results further strengthen the case for mannosidase activity in serum leading to the observed reduction in PGT128 binding. The inhibitory effect of DMJ was less pronounced than with kifunensine, which is consistent with the reported weaker potency of DMJ against alpha-1,2-mannosidases compared to kifunensine^30^.

### **Serum mannosidase may trim oligomannosidic glycoconjugates*in vivo***

**Figure 3:**
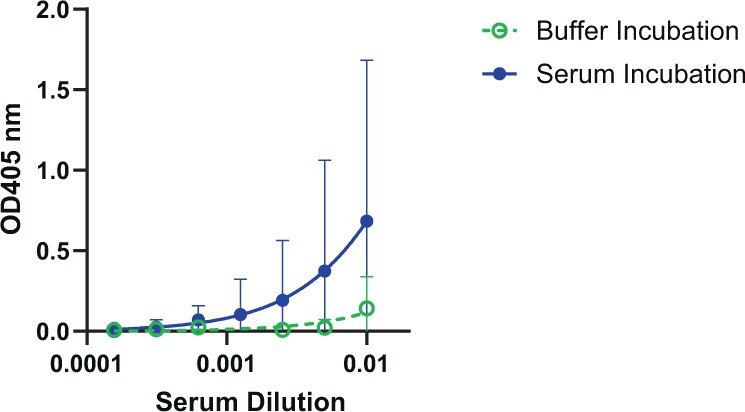
Sera from Trianni mice immunized with the adjuvanted CRM_197_-conjugated version of our oligomannose mimetic bind preferentially to the serum mannosidase-trimmed glycoconjugate. Trianni mice (n = 5) were immunized three times (days 0, 21, 42) with GLA-SE adjuvanted CRM_197_-oligomannoside conjugate NIT211 (pre-made mixture of NIT211_4 (5.9 ligands/CRM_197_) and NIT211_5 (6.5 ligands/CRM_197_). Sera collected on day 49 were assayed for binding to heterologous BSA glycoconjugate (NIT82B)-coated ELISA plates after overnight (24 h) incubation of the glycoconjugate-coated wells with buffer or human serum. Graphs depict geometric mean values for the five serum samples, each assayed in duplicate, with error bars denoting the standard deviation from the mean.

Having confirmed mannosidase activity in serum, we sought also to determine whether serum mannosidase trimming is relevant *in vivo*. To do so, we used sera from Trianni mice that were immunized three times (days 0, 21 and 42) with adjuvanted NIT211, the CRM_197_-conjugated version of our oligomannose mimetic. Trianni mice express a complete repertoire of human variable domains with retention of all mouse constant domains, thus enabling an approximation of potential antibody responses in people. To evaluate the antibody response to the glycoside component without measuring CRM_197_-specific serum antibodies, we assayed sera collected 7 days after the second booster immunization (day 49) for binding to NIT82B, the BSA-conjugated version of the mimetic. The BSA-conjugate was coated onto microtiter wells, then incubated *in situ* overnight with buffer or human serum to evaluate immune serum binding to untrimmed or serum-mannosidase trimmed glycoside, respectively. We found that the sera tended to bind better to the human serum-treated conjugate than to the buffer-treated conjugate (Fig. 3), suggestive of serum mannosidase trimming, at least to some extent, of (synthetic) glycosides conjugates *in vivo* upon immunization.

We did note that only two of the five serum samples bound the buffer-treated BSA conjugate at the serum dilutions assayed here (starting at 1:100) (Supplementary Fig. S3). We confirmed that this binding was glycoside-specific; both serum samples bound negligibly to unconjugated BSA (Supplementary Fig. S4). We nevertheless included all five sera in our assay to determine whether binding to the human serum-treated BSA-conjugate (i.e., trimmed glycoside) would be observed for all sera – which we however did not. The absence of glycoside-binding antibodies in these sera does not appear to be the result of poor immunogenicity of the administered glycoconjugate per se, given the robust serum binding from all five immunized animals to the carrier protein (CRM_197_) of the glycoconjugate used for immunization (Supplementary Fig. S3).

### Serum alpha-mannosidase does not trim oligomannose-type glycans on recombinant envelope glycoprotein trimers

**Figure 4:**
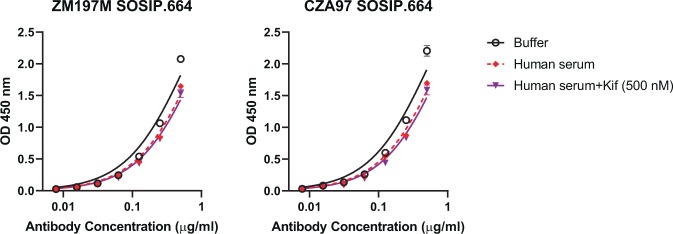
SOSIP trimers are refractory to serum mannosidase trimming. Binding of a murine (IgG2a) version of antibody PGT128 to HIS-tagged SOSIP trimers ZM197M and C.ZA97 absorbed onto Ni^2+^-coated plates (5 µg/ml) was determined following *in situ* overnight incubation with buffer or human serum. Results are from a single experiment.

The results above led us to consider that serum mannosidase might also act on recombinant envelope glycoprotein trimers. So-called SOSIP trimers in particular have gained substantial interest in the last several years as vaccine immunogens^39^. SOSIP trimer glycosylation does not exactly match glycosylation on the native virus spike; SOSIP trimers tend to possess a somewhat higher proportion of oligomannose-type glycans^40,41^, possibly because of quaternary structure differences between SOSIP trimers and the native spike^42,43^. Nevertheless, given the strong interest in using SOSIP and SOSIP-like trimers to elicit bnAbs and progress in the development of other recombinant HIV envelope glycoprotein trimers, determining how exposure to serum mannosidase might affect the presented glycans seems warranted. We found however that there was no significant difference in PGT128 binding to SOSIP trimers ZM197M and C.ZA97^44,45^ that were incubated overnight *in situ* with human serum compared to the buffer-treated controls (Fig. 4). No change in antibody binding was observed upon the addition of kifunensine. Thus, these results show that, in contrast to glycoconjugates, serum alpha-mannosidase does not trim oligomannose presented on recombinant envelope glycoproteins that closely resemble the native spike. Serum mannosidase therefore presumably also does not trim clustered oligomannose on the native glycoprotein spike.

## Discussion

Despite substantial effort, attempts to elicit antibodies to the oligomannose patch on HIV have not truly made much progress to date. Although B cell tolerance is often thought to be the major impediment –and may still be relevant—other factors have received relatively little attention to date. Following from a recent study^20^ in which it was suggested that serum alpha-mannosidase trimming may (also) be an impediment to these efforts, in this report we provide further evidence in support of this thesis. Specifically, our results suggest that serum mannosidase trimming may occur readily following immunization with oligomannosidic glycoconjugates (Fig. 3).

In contrast to our observations with the conjugates, *in vitro* incubation of sera with SOSIP trimers showed no trimming of glycans comprising the oligomannose patch, demonstrating that these oligomannose-type glycans, unlike those presented on glycoconjugates, may be protected from mannosidase trimming. An obvious explanation for this outcome is the presumably denser packing of oligomannose glycans on Env. This outcome might also explain why immunizing rabbits with recombinant yeast expressing an abundance of oligomannose^16,46,47^ or prolonged repetitive immunization of macaques with a recombinant gp140 trimer imparted with an intact oligomannose patch^48^ might yield mannose-specific antibodies with at least some affinity for full-sized oligomannose – albeit insufficient to bind oligomannose when presented normally on HIV. In contrast, immunizing with oligomannose or oligomannose-like glycans displayed on virus-like particles^11,49^, oligomannosidic glycoconjugates^10,12,13,15,17^ or glycopeptides presenting oligomannose^20,28^, on which the oligomannosides might be more accessible to serum mannosidase, has resulted in the elicitation of antibodies that are generally less capable of binding full-sized oligomannose. Note that serum mannosidase trimming is not a phenomenon limited to oligomannosidic glycoconjugates or glycopeptides used to elicit anti-HIV antibodies; high mannose glycoforms are trimmed also on administered therapeutic antibodies *in vivo* by serum mannosidase^22,23^. As such, our results, together with those from Nguyen *et al*.^20^, are fully consistent with past reports of oligomannose-trimming activity in mammalian serum.

The physiological role of serum alpha-mannosidase is not entirely clear. One suggestion^21^ is that it may be responsible for demannosylating serum glycoproteins or plasma membrane glycoproteins, which would explain why oligomannose-type glycans are relatively rare on mammalian cells and tissue^4^ – lest they perhaps inadvertently trigger complement activation via the mannose-binding lectin pathway. Although it is not yet clear where serum mannosidase acts upon an administered antigen *in vivo*, the local concentration of the enzyme or the kinetics of trimming, one possibility is that it is present in transudated plasma entering lymph nodes where it trims antigen poised for immune recognition. Unknown also is whether serum mannosidase levels vary in people; although little variation in serum mannosidase levels has been reported in British people^50^, it is unknown whether the same is true in other populations.

How can we reconcile the development of oligomannose-specific bnAbs with alpha-mannosidase activity in serum? Clearly, the results above with the SOSIP trimers (Fig. 4) indicate that serum mannosidase does not easily trim clustered oligomannose-type glycans on Env, meaning that these glycans would be available for recognition by potential B cells during infection. Indeed, experimental evidence suggests that a sustained presence of envelope glycoprotein with the ‘proper’ glycosylation during weeks or months of infection might trigger the ‘right’ B cells^51,52^. Of course, we do not know which form(s) of the envelope glycoprotein trigger(s) the development of oligomannose-specific bnAbs during infection^53^ or whether the triggered B cells are nominally autoreactive or anergic^54^. Further investigations, perhaps such as done for antibodies to the membrane-proximal region of gp41^55^, may help to answer these questions.

The results presented here might seem to favor the use of SOSIP-based trimers rather than, for example, neoglycoconjugates to elicit bnAbs to (oligomannose-type) glycans on HIV. For example, one study reported on the elicitation of glycan-dependent antibodies resembling precursors of human bnAbs to the oligomannose patch in non-transgenic animals using a ‘designer’ SOSIP-based trimer^56^. Despite potential advantages of SOSIP-based immunogens, one probable disadvantage is glycosylation heterogeneity, which has been shown to be greater at several *N-*glycosylation sites compared to native Env^40^. Synthetic oligomannosides offer an attractive alternative in this regard, enabling production of structurally defined immunogens. Another distinct advantage, and the one on which our glycomimetic approach is largely based, is that synthetic oligomannosides can be designed to overcome potential hindrance in B cell activation; autoreactive B cells can be ‘awakened’ when they are suddenly exposed to cross-reactive antigens in a milieu with the proper immunostimulatory signals^54^.

In conclusion, we confirm and expand here on recent research showing the heretofore unappreciated occurrence of a soluble alpha-1,2-mannosidase in mammalian serum that may be responsible, at least in part, for blunting the ability of past oligomannosidic glycoconjugates to elicit oligomannose-specific antibodies to target HIV. Our results suggest that the mannosidase activity is particularly strong in human and mouse sera; it seems less pronounced in rat and rabbit sera, perhaps explaining why we were previously able to elicit modest levels of oligomannose-specific antibodies in transgenic rats^31^. Identifying one or more approaches that can best overcome this mannosidase activity is an obvious next step. Given the rapidity with which trimming occurs (>90% trimming of Man_9_ to Man_6_ within 24–48 h^21,22^), it is clear that any forthcoming approach will need to be able to prevent or at least minimize mannosidase activity immediately upon administration of the immunogen. Such an approach would need to allow enough time for B cell activation at priming, which for T-dependent primary antibody responses is typically delayed (~2–3 days post-immunization) due to the need for sufficient recruitment of T-cell help for initial B cell activation to occur^57,58^. Therefore, parallel to probing immunization strategies to limit mannosidase trimming, we intend to explore the design of mannosidase-resistant glycosides to provide the highest possible resistance to this unforeseen hindrance. Given that antibodies to the oligomannose patch on HIV-1 are major contributors to neutralization breadth and potency in many HIV-infected individuals^59–61^, we feel that these efforts are worthwhile as part of the broader endeavor of developing an effective HIV vaccine component to elicit bnAbs.

## Methods

### Glycoside synthesis

The soluble heptamannoside mimetic of oligomannose used for these studies, termed NIT68B, was synthesized as described previously^31^.

### General procedure for neoglycoconjugate preparation

The BSA conjugate of ligand NIT68B, with an average loading density of 4 glycosides per BSA molecule, was prepared as reported^31^. The CRM_197_ conjugates were synthesized as described recently^62^ but with minor modifications. The amine ligand NIT68B (3.1 mg, 2.6 μmol) in 0.1 M NaHCO_3_ (1 ml) was vigorously stirred with a solution of thiophosgene (3.9 μl, 51 μmol) in chloroform (1 ml) for 4 h at RT. The organic phase was removed and the aqueous phase extracted four times with CHCl_3_. Traces of organic solvent were removed from the aqueous phase by bubbling a stream of air through the solution. Then a solution of CRM_197_ (1.0 mg, 17.1 nmol) in 0.3 M NaCl/0.1 M NaHCO_3_ (0.5 ml) was added and stirring was continued for 72 h at RT. The solution was passed through an Amicon Spin filter (10 kDa) to remove unreacted ligand and washed with PBS buffer to give a solution of the neoglycoconjugate NIT211_3 (0.7 ml). The protein concentration (0.36 mg/ml) was determined using extinction data obtained with a Nanodrop instrument. Conjugate NIT211_4 was prepared using 4.0 mg (3.3 μmol) NIT68B in 0.1 M NaHCO_3_ (0.5 ml), thiophosgene (10.1 μl, 0.132 mmol) and CRM_197_ (2.0 mg, 34.2 nmol) in 0.3 M NaCl/0.1 M NaHCO_3_ (0.7 ml) and was obtained as a solution of the neoglycoconjugate (1 ml; 1.85 mg/ml). Conjugate NIT211_5 was prepared using 12.0 mg (9.9 μmol) NIT68B in 0.1 M NaHCO_3_ (1.0 ml), thiophosgene (30.4 μl, 0.397 mmol) and CRM_197_ (6.0 mg, 102.7 nmol) in 0.3 M NaCl/0.1 M NaHCO_3_ (1.0 ml) and was obtained as a solution of the neoglycoconjugate (1.5 ml; 3.44 mg/ml). For all conjugates, the amount of conjugated ligand per carrier molecule was determined by MALDI-TOF mass spectrometry. MALDI data showed an average loading density of 4.4 glycosides per BSA molecule^31^ and 3.5–6.5 glycosides per CRM_197_ molecule (Supplementary Fig. S1).

### Antibody expression and purification

Antibodies PGT125, 126, 128 and 130 were expressed from FreeStyle 293 F cells grown in FreeStyle media supplemented with 2% FBS following transfection at a 1:1 ratio of expression plasmids pFUSEss-CLIg-hL2 and pFUSEss-CHIg-hG1 encoding the light and heavy chain, respectively, of each corresponding antibody. The variable light and heavy chains of each antibody were codon-optimized (ATUM) for expression in 293 cells. A murine version of PGT128 was expressed also, using expression plasmids pFUSE2ss-CLIg-mL1 and pFUSEss-CHIg-mG2a encoding the light and heavy chain, respectively, of the antibody. For transfections, plasmids were mixed with either 293fectin, 293-free or ExpiFectamine 293 diluted in Opti-MEM I media; all three agents are optimized for transfection of HEK293-type cells grown in suspension culture. Cell culture supernatants were harvested 6 days after transfection, filtered, concentrated and antibody then purified on individual protein A (human IgG) or protein G (mouse IgG) spin columns as per the recommended protocols. Sample purity was verified by SDS-PAGE and antibody concentration determined on a Nanovue spectrophotometer. Prior to using the murine version of PGT128, we confirmed that it binds antigen just as well as the human version of the antibody (Supplementary Fig. S4).

### Trianni mouse immunizations

Trianni mice (n = 5, mix of male (3) and female (2) animals; 6–8 weeks of age at the start of immunization) were immunized under contract at Antibody Biosolutions (Sunnyvale, CA). For immunization, a ~1:3 mixture consisting of NIT211 derivatives NIT211_4 (5.9 ligands/CRM_197_) and NIT211_5 (6.5 ligands/CRM_197_) was used. The mixed NIT211 conjugate (30 μg, corresponding to ~3 μg of conjugated glycoside) was mixed with an equal amount of glucopyranosyl lipid A in a stable emulsion (GLA-SE), and incubated for 1 h at room temperature before being injected subcutaneously into each animal. The experimental protocol (protocol. no. 1242HS-17) for using mice in this study was approved by the University Animal Care Committee (UACC) of Simon Fraser University and followed relevant guidelines and regulation of animal care. A small bleed was collected from all animals immediately prior to immunization and at 7 days after the boosters at days 21 and 42. The collected blood was left to clot so that serum could be recovered. Serum samples were stored at −20 °C. Once thawed, they were kept at 4 °C.

### ELISA

ELISAs were performed as described previously^31^. For the serum mannosidase trimming experiments, buffer, serum (neat) or serum plus inhibitor were added after the blocking step and incubated overnight (24 h) in a humidified incubator at 37 °C. The next day, the plate was washed and the assay continued as per standard with the primary antibody. For the ELISAs with glycoconjugates or with unconjugated CRM_197_ or BSA, polystyrene microtiter plates were used whereas nickel-coated plates were used for SOSIP trimer ELISAs. p-Nitrophenyl phosphate (p-NPP) or tetramethylbenzidine (TMB) were used as substrate for ELISAs. In the case of TMB, reactions were stopped with sulfuric acid (2 M) and plates read immediately at 450 nm.

### Software/Data analysis

All ELISA data was graphed with Graphpad Prism.

## Supplementary information

Supplementary information.
