# Supplementary material for: Serum alpha-mannosidase as an additional barrier to eliciting oligomannose-specific HIV-1-neutralizing antibodies
Source: Sci Rep. 2020 May 5;10:7582. doi: 10.1038/s41598-020-64500-8 (PMC7200719; doi:10.1038/s41598-020-64500-8)
Supplement: Supplementary file 1 — Supplementary information. [file 41598_2020_64500_MOESM1_ESM.pdf]

SUPPLEMENTAL INFORMATION

Serum alpha-mannosidase as an additional barrier to eliciting oligomannose-specific HIV-1-neutralizing antibodies

Jean-François Bruxelle[1], Tess Kirilenko[1,4], Quratulain Qureshi[2], Naiomi Lu[1,5], Nino Trattnig[3,6], Paul Kosma[3], Ralph Pantophlet[1,2*]*

[1]Faculty of Health Sciences, Simon Fraser University, Burnaby, British Columbia V5A1S6, Canada; [2]Department of Molecular Biology & Biochemistry, Simon Fraser University, Burnaby, British Columbia V5A1S6, Canada; [3]Department of Chemistry, University of Natural Resources and Life Sciences, Vienna A-1190, Austria; [4]Present address: AbCellera Biologics Inc., Vancouver, British Columbia, Canada; [5]Present address: National Collaborating Centre for Infectious Diseases, Winnipeg, Manitoba, Canada; [6]Present address: Department of Chemical Biology and Drug Discovery, Utrecht University, Utrecht, The Netherlands

Figure S1. MALDI-TOF spectra of NIT211 derivatives. *Top:* Compounds NIT211_3, NIT211_4 and NIT211_5 were synthesized in essence as described recently[1], albeit with a few alterations as detailed here in the Methods section. The aminopropyl-equipped oligomannoside was first activated with thiophosgene to form an isothiocyanate intermediate and was then added to a solution of CRM_{197} in buffer to furnish the neoglycoconjugate. A higher dilution of reagents during conjugation resulted in a lower carbohydrate density of the corresponding glycoconjugates. *Bottom:* MALDI-TOF spectra of CRM_{197}, NIT211_3, NIT211_4 and NIT211_5 (top to bottom, respectively). The MALDI-TOF mass spectrometric analysis revealed a ligand:CRM_{197} ratio of 3.5:1 (NIT211_3), 5.9:1 (NIT211_4) and 6.5:1 (NIT211_5).

Figure S2. Inhibition of serum mannosidase trimming by kifunensine and EDTA *in vitro* is titratable and can be achieved with deoxymannojirimycin (DMJ), another alpha-mannosidase inhibitor. (**a**) Titratable inhibition of serum mannosidase trimming *in vitro*. Shown is PGT128 binding to the BSA-conjugated glycoside (at fixed concentration (5 µg/ml)) after in situ overnight incubation of glycoconjugate-coated ELISA plates with rat or mouse containing graded concentrations of kifunensine (left) or EDTA (right). The test concentrations for each inhibitor were performed on a single assay plate to avoid potential plate-to-plate variability. The data show that PGT128 binding increases with increasing inhibitor concentration. (**b**) Inhibition of serum mannosidase trimming by DMJ. Shown is the binding of serially titrated antibody PGT128 to the BSA-conjugated oligomannoside mimetic NIT82B after in situ overnight incubation of a glycoconjugate-

coated (5 µg/ml) ELISA plate with buffer, rat serum, rat serum supplemented with the potent alpha-1,2-mannosidase inhibitor kifunensine (Kif) or rat serum supplemented with the alpha-1,2-mannosidase inhibitor DMJ.

Figure S3. Binding of sera from individual Trianni mice to the BSA-conjugated version of the oligomannose mimetic (termed NIT82B) in comparison to serum binding to CRM_{197}, the protein carrier of our mimetic conjugate used for immunization. *Left:* Sera collected on day 49 from five Trianni mice immunized three times (days 0, 21, 42) with a GLA-SE adjuvanted formulation of our oligomannose mimetic conjugated to CRM_{197} were assayed for binding to a BSA-conjugate of the same glycoside (NIT82B) which had been coated onto ELISA plate wells overnight and subsequently incubated an additional night (24 h) with buffer or with human serum. Each serum was assayed in duplicate, with error bars denoting the standard deviation from the mean. Denoted above each graph is also the sex of each of the five animals. *Right:* ELISA of pre-immune sera (day 0; D0) and immune sera (day 49; D49) from the five individual Trianni mice to the CRM_{197} protein carrier, which was coated onto microtiter plate wells at 5 µg/ml.

Figure S4. Binding of sera from individual Trianni mice to unconjugated BSA. Immune sera collected on day 49 from Trianni mice that exhibited binding to a BSA-conjugate of our oligomannose mimetic (Supplementary Fig. S3) were assayed for binding to unconjugated BSA, which was coated onto microtiter plate wells at 5 µg/ml. Each serum was assayed in duplicate.

Figure S5. Human and mouse versions of bnAb PGT128 bind the oligomannose mimetic NIT82B equivalently. Human IgG1 (blue curve) and mouse IgG2a (red curve) versions of bnAb PGT128 were assayed for binding to BSA glycoconjugate NIT82B coated as solid-phase antigen (5 μg/ml) onto ELISA plate wells.

Reference for supplemental information

1 Trattnig, N., Blaukopf, M., Bruxelle, J.-F., Pantophlet, R. & Kosma, P. Synthesis of an undecasaccharide featuring an oligomannosidic heptasaccharide and a bacterial Kdo-lipid A backbone for eliciting neutralizing antibodies to mammalian oligomannose on the HIV-1 envelope spike. *J Am Chem Soc* **141**, 7946-7954, doi:10.1021/jacs.9b02872 (2019).

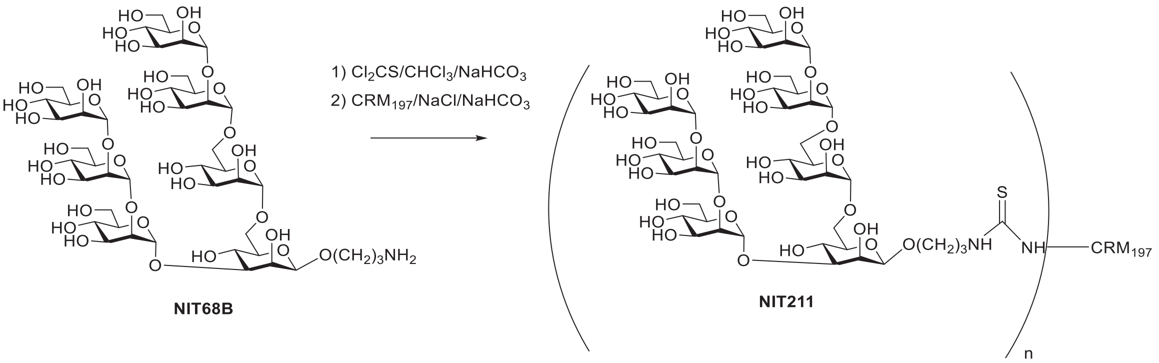
1) Cl2CS/CHCl3/NaHCO3
2) CRM197/NaCl/NaHCO3
O(CH2)3NH2
NIT68B
O(CH2)3NH
NH
CRM197
NIT211
n

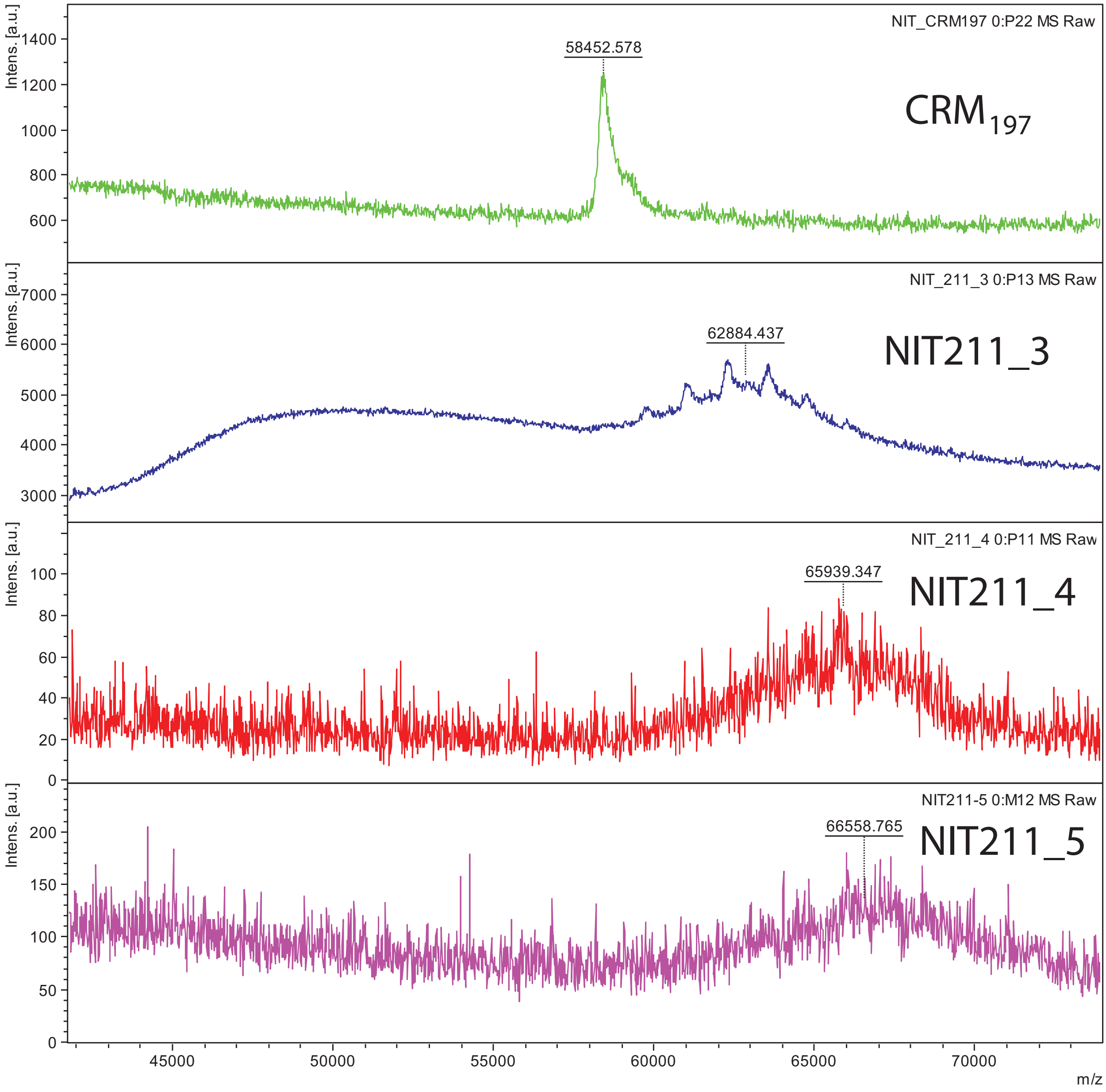
NIT_CRM197 0:P22 MS Raw
58452.578
CRM197
NIT_211_3 0:P13 MS Raw
62884.437
NIT211_3
NIT_211_4 0:P11 MS Raw
65939.347
NIT211_4
NIT211-5 0:M12 MS Raw
66558.765
NIT211_5
Intens. [a.u.]
m/z

Figure S1

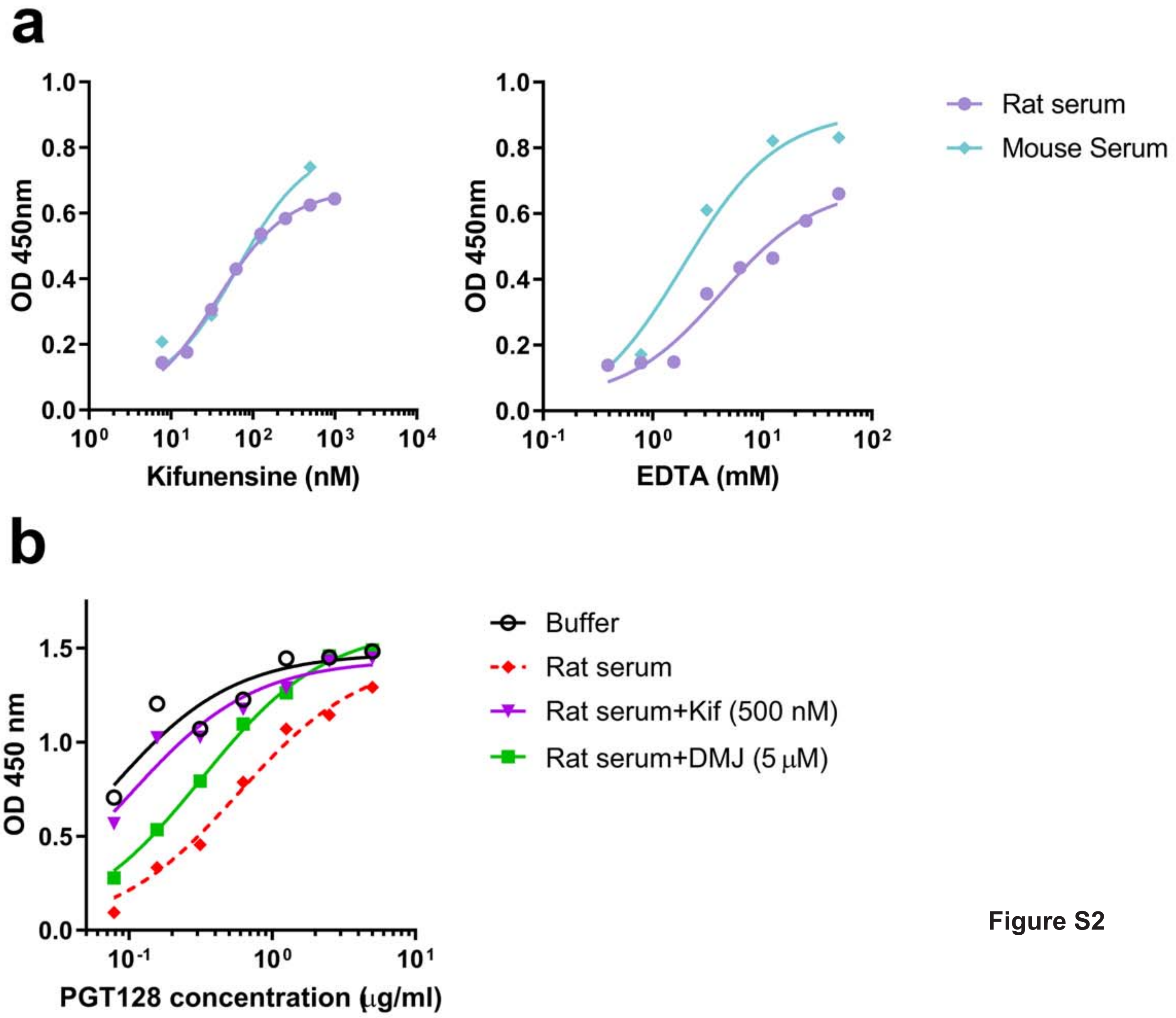
a
OD 450nm
Kifunensine (nM)
EDTA (mM)
Rat serum
Mouse Serum
b
OD 450 nm
PGT128 concentration (μg/ml)
Buffer
Rat serum
Rat serum+Kif (500 nM)
Rat serum+DMJ (5 μM)

Figure S2

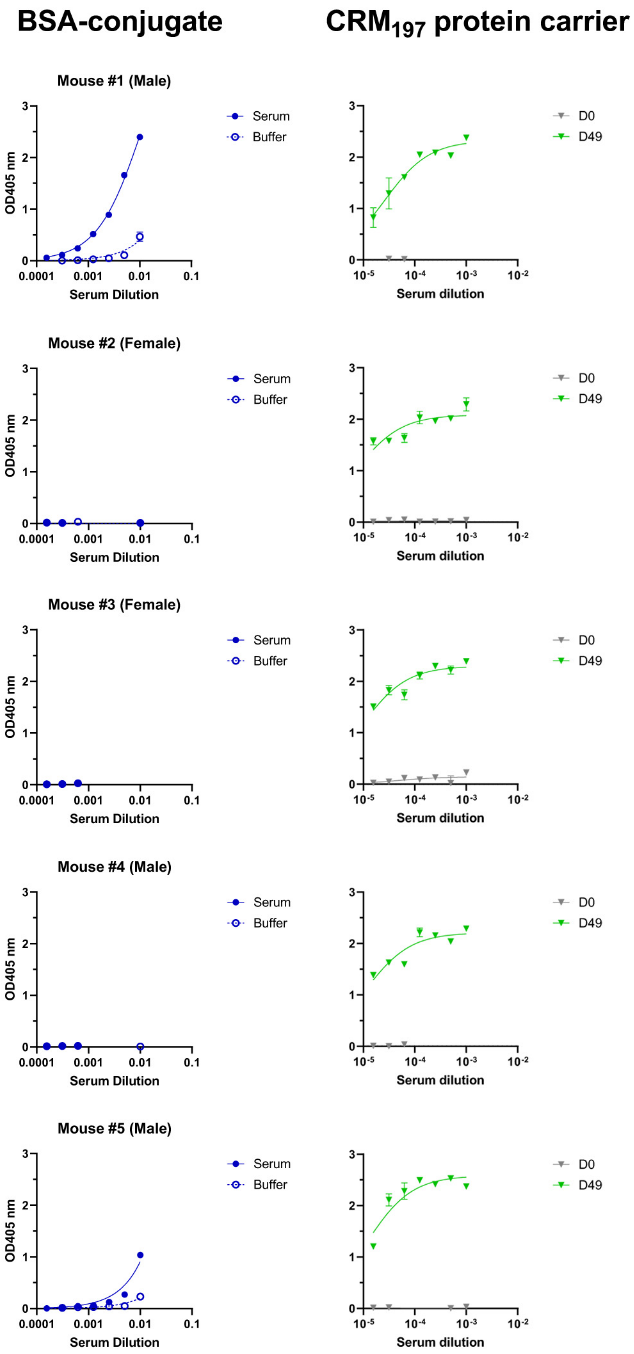

BSA-conjugate
CRM197 protein carrier
Mouse #1 (Male)
Mouse #2 (Female)
Mouse #3 (Female)
Mouse #4 (Male)
Mouse #5 (Male)
OD405 nm
Serum Dilution
Serum dilution
Serum
Buffer
D0
D49
0.0001
0.001
0.01
0.1

Figure S3

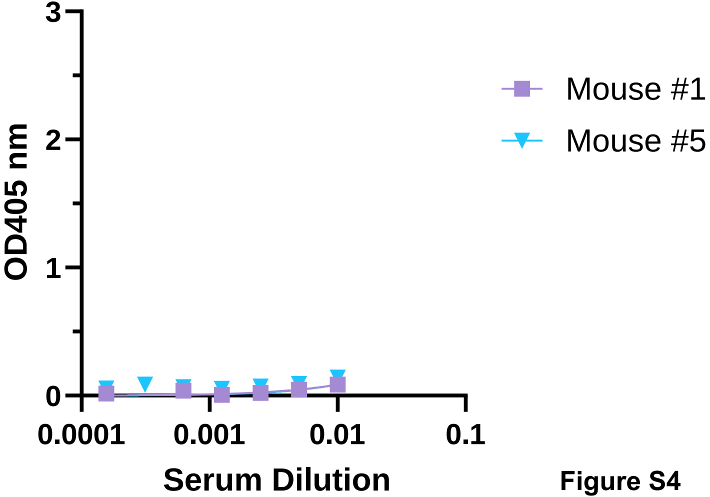
3
2
1
0
OD405 nm
0.0001
0.001
0.01
0.1
Serum Dilution
Mouse #1
Mouse #5

Figure S4

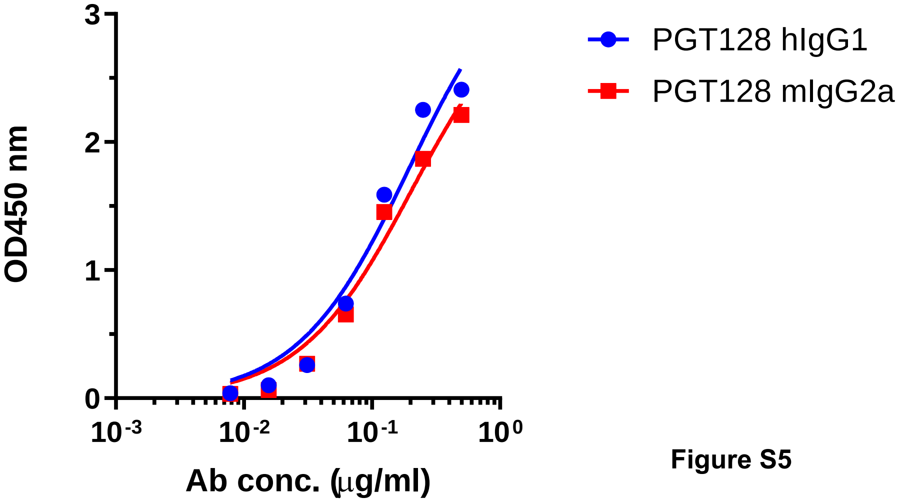

OD450 nm
3
2
1
0
10^-3
10^-2
10^-1
10^0
Ab conc. (μg/ml)
PGT128 hIgG1
PGT128 mIgG2a

Figure S5
